# Corrigendum

**DOI:** 10.1002/ccr3.7864

**Published:** 2023-09-07

Please note that my affiliation has to be corrected as mentioned below and disregard my previous affiliation:

A. Corrected Affiliation:

^1^1‐Team Erevnites, Trivandrum, India.

^2^Virgen Milagrosa University Foundation College of Medicine, San Carlos City, Pangasinan, Philippines.

B. Previous wrong Affiliation:

^1^Team Erevnites, Trivandrum, India.

^2^Computational Cardiovascular Simulations Center, University of Miami, Coral Gables, Florida, USA.

Regards,

Prakash Gupta.

drprakashgupta1993@gmail.com

